# A multidisciplinary, integrated approach for the elimination of schistosomiasis: a longitudinal study in a historically hyper-endemic region in the lower reaches of the Yangtze River, China from 2005 to 2014

**DOI:** 10.1186/s40249-017-0270-x

**Published:** 2017-03-14

**Authors:** Le-Ping Sun, Wei Wang, Yin-Ping Zuo, Qing-Biao Hong, Guang-Lin Du, Yu-Cai Ma, Jian Wang, Guo-Jing Yang, Dao-Jian Zhu, You-Sheng Liang

**Affiliations:** 1 Key Laboratory of National Health and Family Planning Commission on Parasitic Disease Control and Prevention, No. 117 Yangxiang, Meiyuan, Wuxi City, Jiangsu Province 214064 China; 2 Jiangsu Provincial Key Laboratory on Parasites and Vector Control Technology, No. 117 Yangxiang, Meiyuan, Wuxi City, Jiangsu Province 214064 China; 3 Jiangsu Institute of Parasitic Diseases, No. 117 Yangxiang, Meiyuan, Wuxi City, Jiangsu Province 214064 China; 4 Yangzhou Municipal Center for Disease Control and Prevention, No. 36 Yanfu East Road, Yangzhou City, Jiangsu Province 225000 China; 5 Hanjiang District Center for Disease Control and Prevention, Wenhui West Road, Yangzhou City, Jiangsu Province 225000 China

**Keywords:** Schistosomiasis, Elimination, Multidisciplinary approach, Integrated control, Yangtze River, Longitudinal study, China

## Abstract

**Background:**

Although great success has been achieved, schistosomiasis remains a major public health concern in China, and the remaining core endemic regions are concentrated along the middle and lower reaches of the Yangtze River. In this longitudinal study, we evaluated the effectiveness of a multidisciplinary, integrated approach for schistosomiasis elimination in a historically hyper-endemic region in the lower reaches of the Yangtze River, China over the 10-year period from 2005 through 2014.

**Methods:**

A three-step roadmap for schistosomiasis elimination was designed in the study site, and multidisciplinary, integrated interventions were implemented by the health, agriculture, water resources development, land and resources, and forestry sectors from 2005 to 2014, including chemotherapy for infected individuals, health education, management of the source of *Schistosoma japonicum* infection, and intermediate host snail control. The annual number of schistosomiasis patients, *S. japonicum* infection in humans, bovines and *Oncomelania hupensis* snails, and water infectivity were observed to assess the effectiveness of the multidisciplinary, integrated approach for the elimination of schistosomiasis.

**Results:**

There was a tendency towards a gradual decline in both the number of schistosomiasis cases and the prevalence of *S. japonicum* human infection across the study period from 2005 through 2014. No *S. japonicum* human infection was detected since 2012, and no acute infection was seen since 2006. During the study period, no infection was found in bovines, and a 0.03% overall infection rate was observed in *O. hupensis* snails. Since 2009, no infected snails were identified, and the area of both snail habitats and infected snail habitats appeared a reduction over the study period. Following the 3-year multidisciplinary, integrated control, infection control was achieved, and transmission control was achieved after 6-year implementation, with all infected snails and water infectivity eliminated; in addition, the 10-year implementation resulted in interruption of schistosomiasis transmission in the study site in 2014.

**Conclusions:**

The results of the present 10-year longitudinal study demonstrate that the multidisciplinary, integrated approach is effective for the elimination of schistosomiasis as a public health problem in the lower reaches of the Yangtze River, China.

**Electronic supplementary material:**

The online version of this article (doi:10.1186/s40249-017-0270-x) contains supplementary material, which is available to authorized users.

## Multilingual abstract

Please see Additional file 1 for translations of the abstract into the five official working language of the United Nations.

## Background

Schistosomiasis is a neglected tropical disease caused by the blood fluke of the genus *Schistosoma*, which remains a major public health concern worldwide [1]. The disease is estimated to affect 240 million people in 78 countries, with a further 800 million at risk of infection [2]. Worldwide, the total number of disability adjusted life years (DALY) lost due to schistosomiasis is estimated at 1.532 million per year [3], in which 77% are measured in sub-Saharan Africa [4–6]. In addition, meta-analyses estimated 280 000 schistosomiasis-attributable deaths annually in sub-Saharan Africa alone [7, 8]. With the advent of praziquantel in 1970s, a highly effective and lowly toxic schistosomicide with easy administration and competitive cost [9–11], the World Health Organization (WHO) Expert Committee on the Control of Schistosomiasis recommended a shift of the global schistosomiasis control strategy from transmission control to morbidity control [12]. Since then, mass drug administration (MDA) with praziquantel has become the predominant strategy for schistosomiasis control in this wormy world [13–15], and such a strategy has been proved to be effective to greatly reduce both the prevalence and intensity of schistosome infections, which facilitates the progress towards the global elimination of the disease [16–19]. In 2013, the agenda was set for the global schistosomiasis elimination based on the global status of schistosomiasis [20], with 2025 defined as the target date for global elimination as a public health concern [21].

Three major species of the trematode worm *Schistosoma* cause human schistosomiases, *S. mansoni*, *S. haematobium* and *S. japonicum* [1]. Two more species, *S. intercalatum* and *S. mekongi*, are of public health interest but their distribution is geographically limited, while *S. malayensis* is currently not perceived as a human problem even if cases have been reported [22]. *S, japonicum*, *S. mekongi* and *S. malayensis* are zoonoses, the former being the only species in China [1]. Following the control efforts for more than half a century in China [23], notably the implementation of the new integrated strategy with emphasis on the control of infectious sources since 2004 [24–27], the number of cases with *S. japonicum* infection has dramatically reduced from over 11 million at the initiation of the national schistosomiasis control program in 1950s to 77.2 thousand in 2015, and transmission control for schistosomiasis (less than 1% *S. japonicum* infection in humans and bovines, no local acute cases, and no infected snails detected for successive 2 years) has been achieved in the country by 2015 [28]. A two-step roadmap for schistosomiasis elimination was therefore proposed in China in 2015, based on the endemic status of schistosomiasis, with aims to achieve transmission interruption (no local *S. japonicum* infections in humans, bovines and snails for successive 5 years, and establishment of a sensitive, effective surveillance system for schistosomiasis) in the country by 2020 and elimination of the disease (no local *S. japonicum* infections in humans, bovines and snails for successive 5 years after transmission interruption) by 2025 [29].

Currently in China, the remaining core endemic regions are predominantly located along the middle and lower reaches of the Yangtze River, in which more than 92% of the national schistosomiasis patients and over 96% of the total snail habitats are detected [28, 30, 31]. Since 2005, a multidisciplinary, integrated approach was implemented for elimination of schistosomiasis in Yangzhou City, a historically hyper-endemic region for schistosomiasis along the middle and lower reaches of the Yangtze River, China [32]. In this study, we evaluated the effectiveness of the multidisciplinary, integrated approach for schistosomiasis elimination in Yangzhou locating in the lower reaches of the Yangtze River, China over the 10-year period from 2005 through 2014.

## Methods

### Ethical statement

This study was approved by the Ethical Review Committee of Jiangsu Institute of Parasitic Diseases (permission number: IRB00004081). All animal experiments were performed in accordance with the 3R rules for animal experiments and the Guidelines for the Care and Use of Laboratory Animals, and signed informed consent was obtained from all participants included in the study.

### Study site

Yangzhou City is located in the lower reaches of the Yangtze River in the east of China, which has a population of 4.66 million, and covers an area of 6.6 thousand km^2^. Historically, Yangzhou City was highly endemic for *S. japonicum*, and there were 55 townships detected with infections in the city, with more than 300 million people at risk of infection [33]. There were 336 thousand accumulated schistosomiasis cases and accumulated snail habitats of about 0.2 billion m^2^ detected in Yangzhou City [34].

### Roadmap of the multidisciplinary, integrated approach

**Fig. 1:**
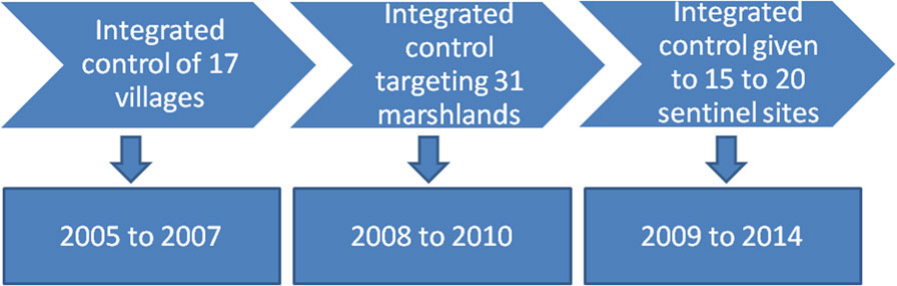
Roadmap of the multidisciplinary, integrated approach for schistosomiasis elimination in Yangzhou City from 2005 to 2014

During the 10-year study period between 2005 and 2014, a three-step roadmap of the multidisciplinary, integrated approach was designed for schistosomiasis elimination in Yangzhou City (Fig. 1). From 2005 to 2007, a total of 17 villages reporting the persistent presence of infected *Oncomelania hupensis* snails or acute schistosomiasis, were selected and subject to the integrated control, including snail control, chemotherapy, health education, replacement of bovines with machines, improved sanitation and access to clean water [35]. Between 2008 and 2010, a total of 31 marshlands with repeated emergence of infected snails were selected and given interventions including prohibition of grazing on marshlands, and snail control with molluscicide treatment and environmental improvment [36]. During the period from 2009 through 2014, 15 to 20 sentinel sites with the detection of positive sentinel mice or frequent human and animal activities were selected in the marshlands along the middle and lower reaches of the Yangtze River and were given a package of interventions consisting of cercarial killing, allocation of excrement collector to boatmen and fishermen, construction of public latrines at assembly centers for mobile boatmen and fishermen and chemotherapy of mobile boatmen and fishermen [37–39].

### Multidisciplinary, integrated approach for schistosomiasis elimination

The multidisciplinary, integrated approach for schistosomiasis elimination consisted of routine control interventions, measures to control the source of *S. japonicum* infection, and integrated snail control. Routine control interventions included chemotherapy for infected individuals, snail survey and control, and health education implemented by health sectors. The measures to control the source of *S. japonicum* infection involved replacing cattle with small farm machines, raising livestock in pens, and examination of schistosomiasis in livestock and chemotherapy for infected livestock implemented by the agriculture sectors, as well as construction of public latrines with three-cell septic tanks and household sanitary toilets completed by the health sectors. Integrated snail control interventions consisted of hardening river banks with concrete, building sluices for prevention of snail spread and digging ditches implemented by the water resources development sectors, constructing fish ponds by the agriculture sectors, land improvement by the departments of land and resources, and building trees in marshlands by the forestry sectors.

### Detection of *S. japonicum* infection in humans and bovines

From 2005 to 2014, 17 villages were selected using the clustering sampling, and all residents living in the enrolled villages were detected for specific IgG antibodies against *S. japonicum* with a dipstick dye immunoassay (DDIA) kit (Wuxi Saide Sci & Tech Development Co., Ltd.; Wuxi, China) during the schistosomiasis non-transmission period in each year [40–42]. Then, all seropositives were subject to miracidium hatching testing for identification of *S. japonicum* infections [43]. At spring and autumn of each year, all bovines in the study villages were detected for *S. japonicum* infection with a miracidium hatching test [44]. The prevalence of *S. japonicum* infection was estimated in both humans and bovines.

### Snail survey

At spring in each year during the period from 2005 through 2014, a snail survey was performed in historical snail habitats using a systematic sampling method [45]. Briefly, a snail collection device, a 0.1 m^2^ square frame made of iron wire, was placed every 20 m along the survey line. All snails within the frame were collected, transferred to the laboratory, counted, and identified for *S. japonicum* infection under a microscope. The area of snail habitats, area with infected snails and snail infection rate were estimated.

### Monitoring of water contamination with *S. japonicum*

Between May and September from 2009 to 2014, *S. japonicum* infection was detected using a mouse bioassay in the sites with detection of acute infections, frequent human and livestock activities, or assembly centers for mobile boatmen and fishermen [46].

### Data management and analysis

A descriptive epidemiological method was employed in this study [47]. All data were processed in Microsoft Excel version 2007 (Microsoft Corporation; Redmond, WA, USA) and all statistical analyses were performed using the statistical software SPSS version 13.0 (SPSS, Inc.; Chicago, IL, USA).

## Results

### Implementation of multidisciplinary integrated interventions

During the 10-year study period from 2005 through 2014, the health departments performed snail survey at 168 542.18 hm^2^, and molluscicide treatment with niclosamide formulations at 32 391.35 hm^2^; in addition, 3 143.645 thousand information, education and communication (IEC) materials were given to high-risk populations, and 1065.2 thousand people received chemotherapy with praziquantel at a single oral dose of 40 mg/kg (Table 1). The health sectors also built 221 public latrines, and 546.6 thousand household sanitary toilets, and the agriculture departments built 5.29 hm^2^ fens to raise livestock, eliminated 402 bovines and treated 101 259 bovines with praziquantel at single dose of 30 mg/kg, aiming to control the source of *S. japonicum* infection (Table 2). Moreover, the water resources development sectors hardened river banks with concrete at 205.25 km, built 68 sluices and dug 182.51 km ditches; the agriculture sectors built 221 fish ponds; the land and resources sectors completed land improvements at 8 704.35 hm^2^, and the forestry sectors built trees at 3 446.06 hm^2^, with attempts to control the intermediate host snails (Table 3).

**Table 1:** Routine control interventions for schistosomiasis implemented in Yangzhou City during the 10-year period from 2005 through 2014

Year	Chemotherapy of humans (thousand persons)	Snail survey (hm^2^)	Molluscicide treatment (hm^2^)	IEC materials (thousand)
2005	95.1	17 027.27	2 403.44	259.925
2006	108.9	17 835.73	2 989.89	255.139
2007	112.7	18 462.18	2 981.72	234.197
2008	95.6	16 771.77	2 746.39	268.596
2009	109.5	15 191.21	2 802.52	368.952
2010	109.2	15 552.25	3 327.29	395.112
2011	113.6	16 515.6	2 902.17	368.153
2012	106.9	17 767.38	3 264.20	320.606
2013	107.6	16 978.76	4 560.88	338.634
2014	106.1	16 440.03	4 412.85	334.331
Total	1 065.2	168 542.18	32 391.35	3 143.645

**Table 2:** Integrated measures to control the source of *S. japonicum* infection implemented in Yangzhou City during the 10-year period from 2005 through 2014

Year	Number of bovine elimination	Building fens to raise livestock (hm^2^)	Chemotherapy of livestock	Construction of public latrines	Construction of household sanitary toilets (thousand)
2005	42	0.04	5 787	0	23
2006	154	0.04	5 087	0	39.2
2007	71	0.04	7 923	21	40.2
2008	36	0.07	8 689	12	49.9
2009	20	0.07	11 701	14	68.6
2010	32	0.96	12 490	24	69.2
2011	11	0.52	12 396	40	72
2012	26	0.54	12 841	36	72.6
2013	10	2.30	11 481	38	72.9
2014	0	0.71	12 864	36	39
Total	402	5.29	101 259	221	546.6

**Table 3:** Integrated snail control interventions implemented in Yangzhou City during the 10-year period from 2005 through 2014

Year	Hardening river banks with concrete (km)	Number of sluices built	Digging ditches (km)	Number of fish ponds built	Land improvement (hm^2^)	Building trees (hm^2^)
2005	1.16	5	4.77	0	393.53	302.02
2006	2.4	0	46.17	0	1 167.25	1 382.02
2007	13.86	39	7.79	21	593.63	310.16
2008	13.12	17	64.47	12	1 787.56	221.24
2009	40.07	4	16.73	14	2 374.52	180.09
2010	14.6	1	7.38	24	1 007.17	526.93
2011	7.25	0	0	40	26.68	286.81
2012	17.49	0	0	36	0	136.74
2013	79.81	0	35.2	38	1 354.01	73.37
2014	15.49	2	0	36	0	26.68
Total	205.25	68	182.51	221	8 704.35	3 446.06

### Overall status of schistosomiasis control from 2005 to 2014

**Fig. 2:**
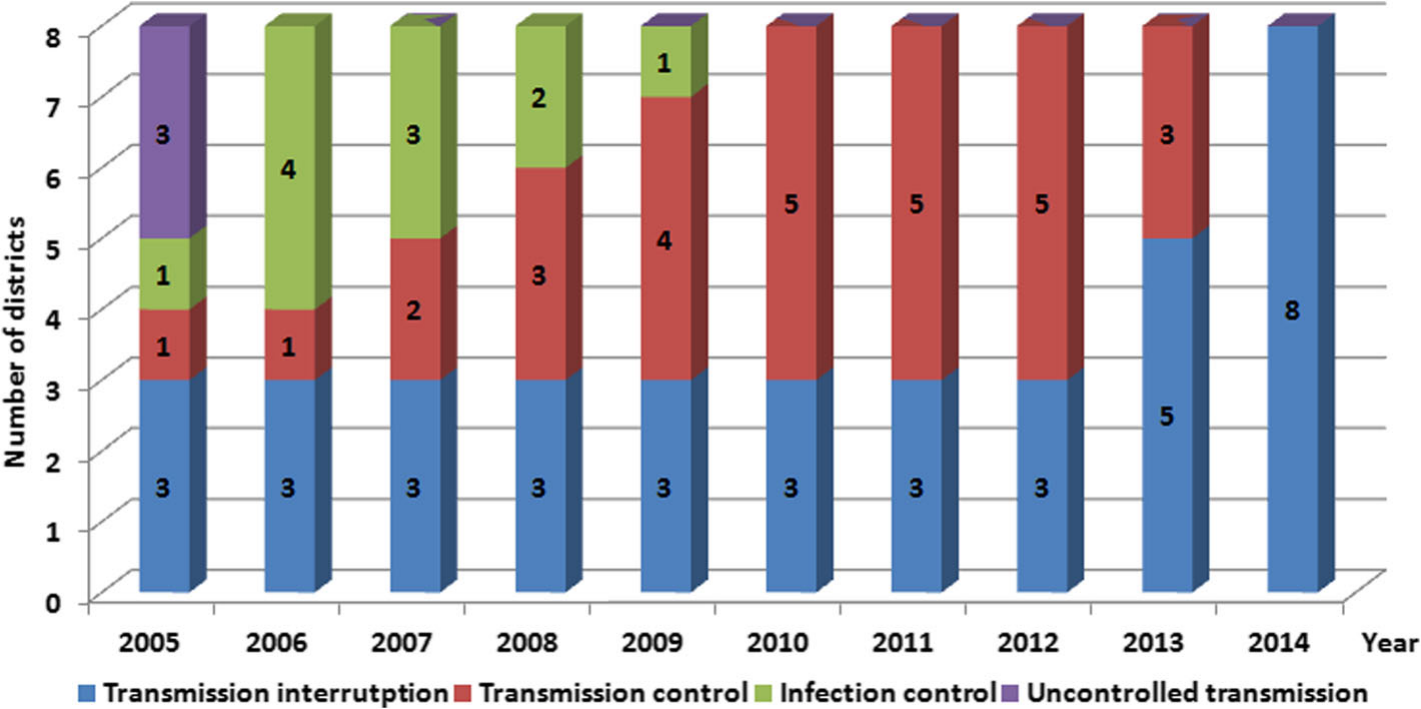
Annual number of districts with infection control, transmission control, transmission interruption and uncontrolled transmission of schistosomiasis in Yangzhou City from 2005 to 2014

**Fig. 3:**
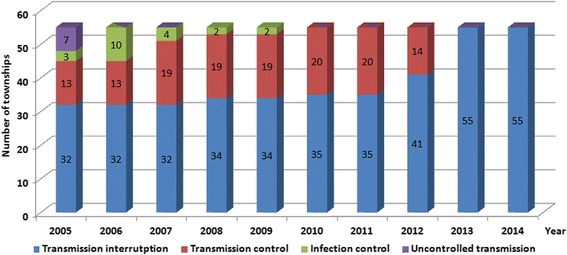
Annual number of townships with infection control, transmission control, transmission interruption and uncontrolled transmission of schistosomiasis in Yangzhou City from 2005 to 2014

In 2005, there were three out of the eight schistosomiasis-endemic districts and seven out of the 55 endemic townships with uncontrolled transmission in Yangzhou City. Following the implementation of the multidisciplinary, integrated approach, infection control of schistosomiasis (less than 5% *S. japonicum* infection in humans and bovines, and no outbreak of acute schistosomiasis) was achieved in the study site in 2007, transmission control achieved in 2010, and transmission interruption achieved in 2014 (Figs. 2 and 3).

### *S. japonicum* infection in humans and bovines from 2005 to 2014

**Fig. 4:**
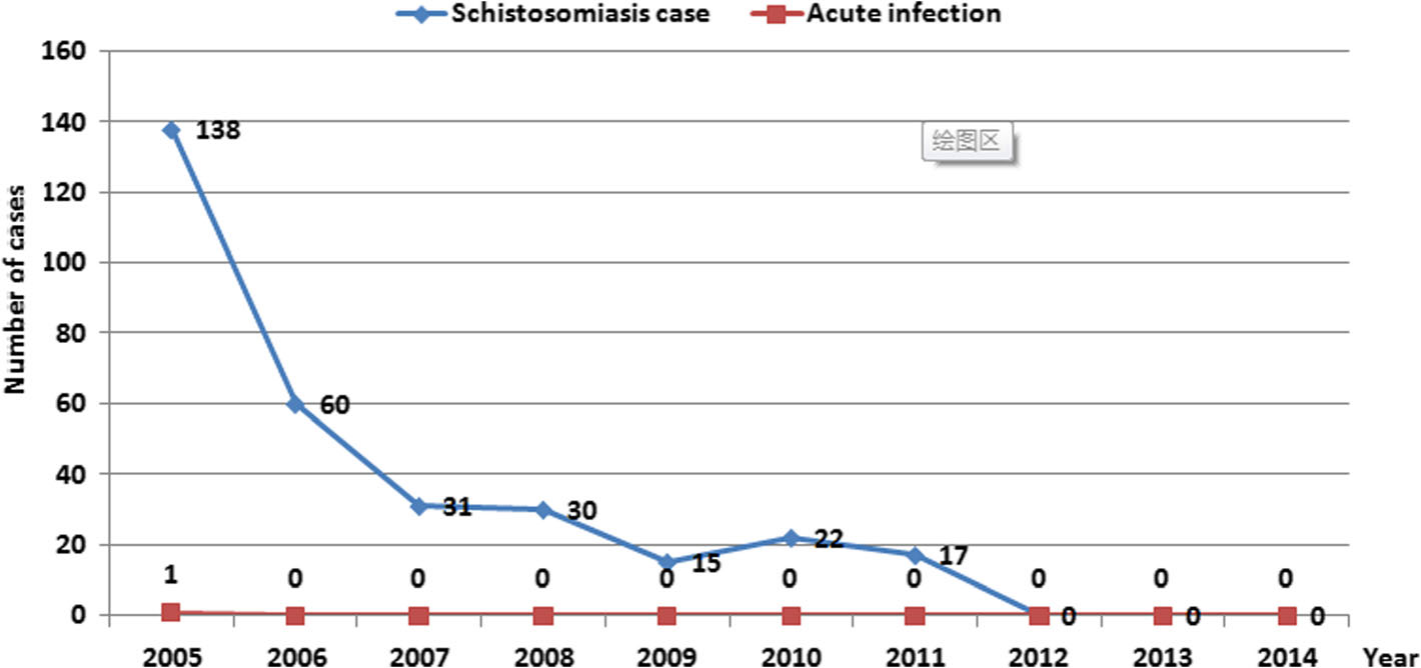
Annual number of schistosomiasis cases and acute infections in Yangzhou City from 2005 to 2014

**Fig. 5:**
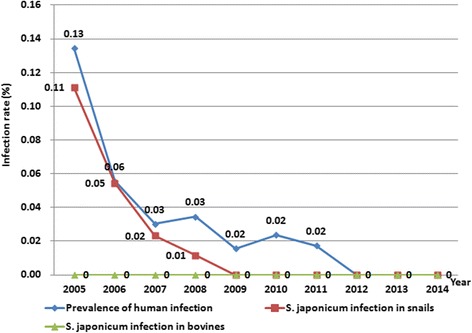
Rates of *S. japonicum* infection in humans, bovines and snails in Yangzhou City 2005 to 2014

During the study period, a total of 954477 individuals received serological examinations with DDIA, and all seropositives were then subject to the miracidium hatching test. Finally, a total of 313 egg-positive individuals were identified, with 0.03% overall prevalence of *S. japonicum* infection. Since 2012, no *S. japonicum* human infection was detected, and no acute infection was seen since 2006. There was a tendency towards a gradual decline seen in both the number of schistosomiasis cases and the prevalence of *S. japonicum* infection across the study period from 2005 through 2014 (Figs. 4 and 5). A total of 4 481 bovines were detected for *S. japonicum* infection with the miracidium hatching test between 2005 and 2014, and no infection was identified (Fig. 5).

### Outcomes of snail control

**Fig. 6:**
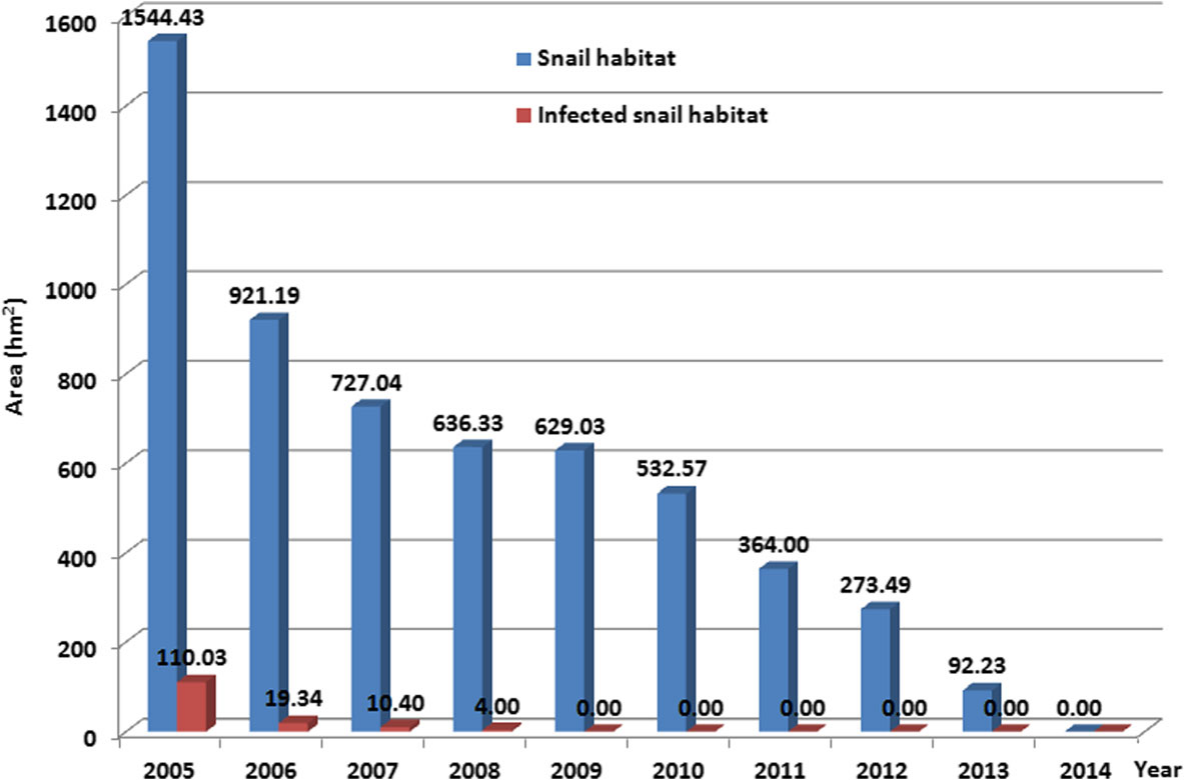
Annual area of snail habitats and infected snail habitats in Yangzhou City 2005 to 2014

From 2005 to 2014, integrated snail control was employed, which were implemented by health, water resources development, agriculture, land and resources, and forestry sectors. During the 10-year study period, a total of 282079 snails were captured and examined for *S. japonicum* infection, and 95 snails were identified with infection, with a 0.03% overall infection rate. Since 2009, no infected snails were identified (Fig. 5). In addition, the area of both snail habitats and infected snail habitats appeared a reduction over the study period, and infected snail habitats were eliminated in the study site since 2009 (Fig. 6).

### Water infectivity

During the period from 2009 through 2014, a total of 351 sentinel sites were assigned, and 5 sites were identified positive, with an overall positive rate of 1.42%. Of the totally 6 507 mice examined, 14 mice were detected positive, with a 0.22% overall positive rate. Since 2010, neither positive sites nor positive mice were detected in the study site (Table 4).

**Table 4:** Annual findings of water contamination with *S. japonicum* from 2009 through 2014

Year	Number of sites investigated	Number of positive sites	Positive rate in the study site (%)	Number of mice examined	Number of positive mice	Positive rate in mice (%)
2009	55	5	9.09	1 074	14	1.3
2010	55	0	0	954	0	0
2011	60	0	0	1 128	0	0
2012	70	0	0	1 177	0	0
2013	36	0	0	696	0	0
2014	75	0	0	1 477	0	0
Total	351	5	1.42	6 506	14	0.22

## Discussion

Schistosomiasis has been widely recognized as a disease that is socially determined [48], and the transmission and control of this disease of poverty is considered to be strongly linked to multiple social, economic and behavioral factors [49–53]. In addition, it is indicated that an integrated, multi-sectoral control approach is necessary for sustainable schistosomiasis control and progressively moving towards elimination [54].

The national schistosomiasis control program was initiated in China at early 1950s [55–57]. At the initial stage of the national schistosomiasis control program, extensive farming and undeveloped water conservancy facilities resulted in the wide distribution of the intermediate host snails. Farmers lived close to water, and had a high possibility to get *S. japonicum* infection [58–60]. With the socio-economic development, the increase in the frequency of human activities may also lead to a rise in the likelihood of the parasite infection [61]. Based on the epidemiological profiles and status of schistosomiasis and the national social and economic situation, integrated strategies have been proposed for schistosomiasis control in China [62], aiming to eliminate this public health concern in the country through integration of multi-sectoral resources and multidisciplinary tools [63–65]. Until late 1990s, schistosomiasis elimination had been achieved in 5 out of the 12 endemic provinces in China [66–68]. Notably, the wide implementation of the integrated strategy with emphasis on infectious source control throughout the main endemic foci of China since 2004 has been proved to greatly facilitate the progress towards the elimination of schistosomiasis in the country [26, 27, 69–80].

Currently, China is moving from transmission control towards transmission interruption and elimination of schistosomiasis [29], and the schistosomiasis control programs require a shift from “extensive control” to “precision control” [81]. Implementation of a highly effective and precise roadmap and approach, which tailors to the intensity of transmission, has been recognized as a key factor that determines the sustainable schistosomiasis control [82–84].

In this study, a three-step roadmap for schistosomiasis elimination was designed in Yangzhou City, a historically hyper-endemic region in the lower reaches of the Yangtze River, China, and multi-sectoral resources were mobilized through integration of multidisciplinary, integrated interventions implemented by the health, agriculture, water resources development, land and resources, and forestry sectors, including chemotherapy for infected individuals, health education, integrated control of the source of *S. japonicum* infection, and integrated snail control. During the 10-year study period from 2005 through 2014, the number of schistosomiasis cases appeared a tendency towards a gradual decline year by year, and the infection rates in both humans and snails, as well as the area of both snail habitats and infected snail habitats showed a reduction over the study period. Following 3-year multidisciplinary, integrated control, infection control was achieved, and transmission control was achieved after 6 years, with all infected snails and water infectivity eliminated in the study site; in addition, the 10-year implementation of this multidisciplinary, integrated approach resulted in interruption of schistosomiasis transmission in the study site in 2014. Our data indicate that the multidisciplinary, integrated approach mobilizing multi-sectoral resources is an effective approach leading to schistosomiasis elimination in marshland and lake regions.

Currently, the Kato-Katz technique and miracidium hatching test remain the gold standard for the diagnosis of *S. japonicum* human infection [85]. However, these two techniques exhibit a high missing rate in detecting *S. japonicum* infections, notably in low-intensity regions [43]. Recently, a variety of immunodiagnostics and molecular biological assays have been developed, which shows a high sensitivity and specificity for the detection of *S. japonicum* human infections [86–88]. A combination of parasitologic techniques and immunodiagnostics/molecular biological assays may greatly reduce the missing rate for detecting *S. japonicum* infections, which facilitates the national schistosomiasis elimination program in China.

Of the six types of human schistosomiasis, the transmission cycle and epidemiological factors linked to schistosomiasis japonica seem more complicated than other five types [1]. *O. hupensis* snail, the only intermediate host of *S. japonicum*, is widely distributed along the Yangtze River basin, and the annual flood results in extensive snail spread in the middle and lower reaches of the Yangtze River, China [45]. In addition to humans, over 40 species of wild and domestic animals may serve as reservoir hosts for *S. japonicum* [9], which complicates the control efforts [89–92]. Currently, China is facing rapid socio-economic development and large eco-environmental changes. It is suggested that the schistosomiasis elimination program should be developed tailored to the socio-economic development plan and the natural and environmental factors affecting the transmission of schistosomiasis in the endemic regions. In addition, a highly effective, sensitive surveillance-response system is of great importance for the rapid identification and elimination of the source of *S. japonicum* infection, which is effective to sustain the control achievements and facilitate the progress towards schistosomiasis elimination [93–96].

## Conclusions

The current study presents a multidisciplinary, integrated approach for schistosomiasis elimination in the lower reaches of the Yangtze River, China, and results of the 10-year longitudinal study between 2005 and 2014 demonstrate that this approach is effective to eliminate schistosomiasis as a public health problem in the marshland and lake regions, which provides new insights into the development of the national schistosomiasis elimination program in China. Currently, China is transferring its successful experiences on schistosomiasis control to Southeast Asia and Africa [97, 98], our multidisciplinary, integrated approach may provide valuable experiences for the global schistosomiasis elimination programs.

## Additional file

Additional file 1: Multilingual abstract in the five official working languages of the United Nations. (PDF 1100 kb)

## Abbreviations

DALY: Disability adjusted life years
DDIA: Dipstick dye immunoassay
IEC: Information, education and communication
MDA: Mass drug administration
WHO: World Health Organization
