# Supplementary material for: A multidisciplinary, integrated approach for the elimination of schistosomiasis: a longitudinal study in a historically hyper-endemic region in the lower reaches of the Yangtze River, China from 2005 to 2014
Source: Infect Dis Poverty. 2017 Mar 14;6:56. doi: 10.1186/s40249-017-0270-x (PMC5348877; doi:10.1186/s40249-017-0270-x)
Supplement: Additional file 1: — Multilingual abstract in the five official working languages of the United Nations. (PDF 1100 kb) [file 40249_2017_270_MOESM1_ESM.pdf]

Translation of the abstract into the five official working languages of the United Nations

نهجٌ متكاملٌ متعددُ التخصصات للقضاء على البلهارسيا: دراسةٌ طوليةٌ في منطقة تاريخية موبوءة في أسفل مجرى نهر اليانغتسى، الصين 2005-2014

لي- بينغ سون، وي وانغ، يين- بينغ زو، كينغ-باو هونغ، غوانغ- لين دو، يو- كاي ما، جيان وانغ، غيو-غينغ يانغ، داو-جيان زهو، يو-شينغ ليانغ

الملخص

خلفية: على الرغم من النجاحات الواسعة في هذا المجال، إلا أنَّ البلهارسيا لا زال مشكلةً صحيةً عامةً ورئيسيةً في الصين وفي باقي المناطق الرئيسية الموبوءة الأساسية على طول الروافد الوسطى والدنيا من نهر اليانغتسى. في هذه الدراسة الطولية، قُمنا بتقييم فعاليةِ نهجٍ متكاملٍ متعددِ التخصصات للقضاء على البلهارسيا في منطقةٍ تاريخيةٍ موبوءة في أسفل مجرى نهر اليانغتسى، الصين 2005-2014.

الطرق: تم تصميم خارطة طريق ثلاثية الخطوات للحد من البلهارسيا في موقع الدراسة وتم توظيف تدخلات متكاملة متعددة التخصصات من قسم تطوير الموارد الصحية والزراعية والمائية، والأراضي والموارد، وقطاعات الغابات من 2005- 2014، بما فيها العلاج الكيميائي للأفراد المصابين، والتثقيف الصحي، وإدارة مصدر داء البلهارسيات اليابانية ومكافحة القوقع العائل الوسيط. تم فحص الأعداد السنوية من مرضى البلهارسيا، وإصابات داء البلهارسيات اليابانية لدى الإنسان والبقر وحلزون "القوتمة الخوبية"، وعدوى المياه لتقييم فعالية النهج المتكامل متعدد التخصصات للقضاء على البلهارسيا.

النتائج: كان هناك توجه نحو انخفاض تدريجي في كلٍّ من عدد حالات البلهارسيا وفي انتشار داء البلهارسيات اليابانية بين البشر طوال مدة الدراسة من عام 2005 وحتى عام 2014. تم رصد عدد المصابين بداء البلهارسيات اليابانية بين البشر منذ عام 2012 ولم نلحظ وجود أي عدوى حادة منذ 2016. خلال مدة الدراسة، لم يتم إيجاد حالات عدوى لدى البقر مع ملاحظة معدل عام لعدوى حلزون "القوتمة الخوبية" بما نسبته 0.03%. لم يتم رصد أي حلزونات مصابة منذ 2009 وشهدت منطقة مواطن الحلزون ومواطن الحلزون المصاب على حد سواء انخفاضاً خلال فترة الدراسة. بعد المكافحة المتكاملة متعددة التخصصات التي دامت لـ 3 سنوات، تم إجراء مكافحة انتشار للعدوى ومكافحة للانتقال بعد 6 سنوات من التنفيذ أسفرَ عن تقليل أعداد الحلزونات المصابة وعدوى الماء بالإضافة إلى ما أسهم به تنفيذ الـ 10 أعوام في وقف انتقال البلهارسيا في موقع الدراسة في 2014.

الخاتمة: توضِّح نتائج الدراسة الطولية الحالية ذات العشر سنوات أنَّ النهج المتكامل متعدد التخصصات فعالٌ في التقليل من البلهارسيا بوصفها مشكلةً صحيةً عامةً في الروافد السفلية من نهر اليانغتسي، الصين.

Translated from English version into Arabic by Ghaida1990, through

TRANSLATORS WITHOUT BORDERS

一种用于血吸虫病消除的多学科综合防治策略：2005~2014 年中国长江下游一个历史重度流行区的纵向观察

孙乐平，汪伟，左引萍，洪青标，杜广林，马玉才，王建，杨国静，朱道建，梁幼生

摘要：

研究背景：尽管中国血吸虫病防治工作取得了举世瞩目的巨大成就，但日本血吸虫病的危害仍然是中国的重要公共卫生问题之一。目前，中国的血吸虫病流行区主要集中在长江中下游的江湖洲滩地区。本研究旨在评价一种多学科综合防治策略对中国长江下游一个历史重度流行区血吸虫病消除的作用与效果。

方法：选择长江下游历史重度流行区扬州市作为试验现场。2005~2014 年，按照疫情控制、传播控制和传播阻断 3 个阶段，实施渐进式的“重点村→重点环境→重点水域”血防综合治理路径，并由卫生、农业、水利、国土资源和林业等部门实施血防综合治理，包括传染源综合管理、有螺环境综合治理、重点人畜同步化疗、重点人群血防健教和高危环境化学灭螺等措施。连续开展人畜病情、螺情和水体感染性监测，综合评价该多学科综合防

治措施用于血吸虫病消除的效果。
结果: 2005~2014 年，扬州市血吸虫病人数和居民血吸虫感染率呈逐年下降趋势，自 2012 年后未发现血吸虫病人，自 2006 年后未发生急性血吸虫感染，未发现血吸虫感染病牛。研究初期（2005 年），钉螺感染率为 0.11%，自 2009 年后未发现感染性钉螺，有螺面积和感染性钉螺面积均呈逐年下降趋势。扬州市实施多学科综合防治 3 年后，控制了急性血吸虫病发生，达到了血吸虫病疫情控制标准；6 年后消除了感染性钉螺，达到了传播控制标准；8 年后消除了钉螺和水体血吸虫感染性，全市于 2014 年达到了血吸虫病传播阻断标准。
结论: 10 年纵向观察结果表明，多学科综合防治可有效消除中国长江下游江滩型地区的血吸虫病危害。

Translated from English version into Chinese by Wei Wang

Une approche pluridisciplinaire et intégrée pour l'éradication de la schistosomiase : étude longitudinale dans une région historiquement hyper-endémique dans le cours inférieur du fleuve Yangtsé, en Chine 2005-2014

Le-Ping Sun, Wei Wang, Yin-Ping Zuo, Qing-Biao Hong, Guang-Lin Du, Yu-Cai Ma, Jian Wang, Guo Jing-Yang, Dao-Jian Zhu, You-Sheng Liang

Résumé
Contexte: de grandes avancées ont été enregistrées. Cependant, la schistosomiase reste un problème majeur de santé publique en Chine, et les régions endémiques de base restantes sont concentrées le long des cours moyen et inférieur du fleuve Yangtsé. Dans cette étude longitudinale, nous avons évalué l'efficacité d'une approche pluridisciplinaire et intégrée pour l'éradiction de la schistosomiase dans une région historiquement hyper-endémique - le cours inférieur de la rivière Yangtsé en Chine, au cours de la période de 10 ans de 2005 à 2014.
Méthodologie: Une feuille de route en trois étapes pour l'éradication de la schistosomiase a été conçue sur le site de l'étude, et des interventions intégrées et pluridisciplinaires ont été mises en œuvre par les responsables de la santé, de l'agriculture, du développement des ressources hydriques, des domaines et des ressources minières, ainsi que ceux du secteur forestier de 2005 à 2014. Par ailleurs, une chimiothérapie a été organisée pour les personnes infectées, une sensibilisation sur la santé, la gestion de la source d'infection de *Schistosoma japonicum* et le contrôle intermédiaire des gastéropodes porteurs. Les chiffres annuels de patients et de bovins atteints d'une infection de schistosomiase *S. japonicum* , de gastropodes *Oncomelania hupensis* et d'infectiosité de source hydrique ont été observés afin d'évaluer l'efficacité de l'approche pluridisciplinaire et intégrée pour l'éradication de la schistosomiase.
Résultats: Ils montraient une tendance en baisse progressive à la fois du nombre de cas de schistosomiase et de la prévalence du *S. japonicum* d'infection humaine à travers la période d'étude de 2005 à 2014. Aucune *S. japonicum* d'infection humaine n'a été détectée depuis 2012, et aucune infection aiguë n'a été observée depuis 2006. Par ailleurs, au cours de la période d'étude, aucune infection bovine n'a été trouvée, et un taux global d'infection de gastéropode *O. hupensis* de 0,03% a été observé. Depuis 2009, aucun gastéropode infecté n'a été identifié et leur zone d'habitat ainsi que celle de ceux infectés semblait avoir réduit au cours de la période de l'étude. 3 ans après la mise en oeuvre de l'étude pluridisciplinaire et intégrée, le taux d'infection était déjà sous contrôle et celui de la transmission de la maladie après 6 ans avec une éradication complète des gastéropodes infectés et de l'infectuosité de l'eau ; en outre, la mise en œuvre de 10 ans a entraîné l'interruption de la transmission de la schistosomiase sur le site de l'étude en 2014.
Conclusions: Les résultats de la présente étude longitudinale de 10 ans démontrent que l'approche pluridisciplinaire et intégrée s'avère efficace pour l'éradication de la schistosomiase en tant que problème de santé publique dans le cours inférieur du fleuve Yangtze, en Chine.

Translated from English version into French by simonyetna, through

TRANSLATORS
WITHOUT BORDERS

Многопрофильный, комплексный подход к борьбе с шистосомозом: продольное исследование, проводимое в исторически гиперэндемическом регионе, расположенном в нижнем течении реки Янцзы, Китай, и охватывающее период с 2005 по 2014 гг.

Ле-Пин Сан, Вэй Ван, Инь-Пин Цзо, Цин-Бяо Хон, Гуан-Лин Ду, Ю-Цай Ма, Цзянь Ван, Го-Цзин Ян, Дао-Цзянь Чжу, Ю-Шэн Лян

Краткое изложение

История вопроса. Несмотря на то, что ученым удалось достигнуть заметных успехов, шистосомоз остается одной из основных проблем общественного здравоохранения в Китае, в то время как остальные ключевые эндемические регионы сосредоточены вдоль среднего и нижнего течения реки Янцзы. В этом продольном исследовании мы оценивали эффективность многопрофильного, комплексного подхода к борьбе с шистосомозом в исторически гиперэндемическом регионе, расположенном в нижнем течении реки Янцзы, Китай, в течение более чем 10-летнего периода с 2005 по 2014 гг.

Методика. В период с 2005 по 2014 гг. в исследовательском центре была разработана трехступенчатая дорожная карта по борьбе с шистосомозом, в то время как в секторах здравоохранения, сельского хозяйства, освоения водных ресурсов, земельных и природных ресурсов, а также в лесном секторе были проведены многопрофильные, комплексные мероприятия, включая химиотерапию инфицированных лиц, санитарное просвещение, управление источником распространения инфекции *Schistosoma japonicum* и контроль популяции промежуточного хозяина паразита — улитки. Проводился мониторинг ежегодного числа больных шистосомозом, людей, крупного рогатого скота и улиток *Oncomelania hupensis,* инфицированных *C. japonicum,* а также степени загрязненности воды с целью оценки эффективности многопрофильного, комплексного подхода к борьбе с шистосомозом.

Результаты. В ходе периода исследования, с 2005 по 2014 гг., наблюдалась тенденция к постепенному снижению как количества случаев шистосомоза, так и распространенности инфекции *S. japonicum* у людей. С 2012 года не было зафиксировано ни одного случая заражения *S. japonicum* человека, а с 2006 года отсутствует информация об острых случаях заболевания. В ходе исследования не было зафиксировано ни одного случая инфицирования крупного рогатого скота, в то время как общий уровень инфицирования улиток *O. hupensis* составил 0,03%. С 2009 года не было выявлено ни одного случая заражения улиток, а площадь как постоянных мест обитания улиток, так и зараженных мест обитания улиток в ходе исследования лишь сократилась. После реализации трехлетних мероприятий по обеспечению многопрофильного комплексного контроля удалось установить контроль над распространением и передачей инфекции (в последнем случае потребовалось 6 лет), включая полную ликвидацию всех зараженных улиток и нормализацию уровня загрязненности воды; кроме того, проводимые в течение 10 лет мероприятия привели к прекращению передачи в шистосомоза в 2014 году.

Выводы. Результаты настоящего продольного 10-летнего исследования подтверждают, что многопрофильный, комплексный подход эффективен для ликвидации шистосомоза как проблемы общественного здравоохранения в регионах Китая, расположенных в нижнем течении реки Янцзы.

Translated from English version into Russian by Irina Zayonchkovskaya, through

Un enfoque multidisciplinario e integrado sobre la eliminación de la esquistosomiasis: estudio longitudinal en una región históricamente hiperendémica del curso inferior del río Yangtze, China 2005-2014

Le Ping-Sun, Wang Wei, Yin-Ping Zuo, Qing-Hong Biao, Guang-Lin Du, Yu-Cai Ma, Wang Jian, Guo Jing-Yang, Dao-Jian Zhu, Te-Sheng Liang

Resumen

Antecedentes: a pesar de los grandes avances, la esquistosomiasis sigue siendo un problema de salud pública importante en China. El nucleo de las regiones que todavía son endémicas se concentra a lo largo del curso medio y bajo del río Yangtze. En este estudio longitudinal, se evaluó la eficacia de un enfoque multidisciplinario e integrado para eliminar la esquistosomiasis en una región históricamente hiperendémica del curso inferior del río Yangtze, China, a lo largo de 10 años; entre 2005 y 2014.

Métodos: se diseñó, en el sitio de estudio, una hoja de ruta de tres pasos para eliminar la esquistosomiasis, y los sectores forestales, de salud, agricultura, desarrollo de recursos hídricos y de suelo y recursos implementaron, entre 2005 y 2014, intervenciones integradas. Éstas incluyeron la quimioterapia para personas afectadas, la educación sobre salud, el manejo de la fuente de infección de *Schistosoma japonicum*, y el control estratégico de caracoles huéspedes. Se observó la cantidad anual de pacientes con esquistosomiasis, la infección por *S. japonicum* en humanos, bovinos y caracoles *Oncomelania hupensis* y la infectividad del agua, a fin de evaluar la eficacia del enfoque multidisciplinario e integrado para eliminar la esquistosomiasis.

Resultados: se observó una tendencia hacia una disminución gradual tanto en la cantidad de casos de esquistosomiasis como en la prevalencia de infección por *S. japonicum* en humanos, a lo largo del período de estudio entre 2005 y 2014. No se detectó ninguna infección por *S. japonicum* en humanos desde 2012, y no hay registros de infecciones agudas desde 2006. Durante el período de estudio, no se encontró la infección en bovinos, y se observó una tasa general de infección del 0,03 % en caracoles *O. hupensis*. Desde el año 2009, no se identificaron caracoles infectados, y tanto el área de hábitat de caracoles como la de hábitat de caracoles infectados se vieron reducidas a lo largo del periodo de estudio. Tras 3 años de control multidisciplinario e integrado, se logró controlar la infección. Tras un periodo de implementación de 6 años, se logró controlar la transmisión, y se logró la eliminación de todos los caracoles infectados y de la infectividad del agua. Además, luego de 10 años de implementación, se logró interrumpir la transmisión en el sitio de estudio en 2014.

Conclusiones: los resultados del presente estudio longitudinal de 10 años demuestran que el enfoque multidisciplinario e integrado es eficaz para la eliminación de la esquisomiasis como problema de salud pública en el curso inferior del río Yangtze, China.

Translated from English version into Spanish by Maria Paula Gorgone, through
